# Primary Retroperitoneal Lymph Node Dissection as Treatment for Low-volume Metastatic Seminoma in a Population-based Cohort: The Swedish Norwegian Testicular Cancer Group Experience

**DOI:** 10.1016/j.euros.2024.05.006

**Published:** 2024-06-11

**Authors:** Anna Thor, Helene F.S. Negaard, Anna Grenabo Bergdahl, Bjarte Almås, Signe Melsen Larsen, Per-Olof Lundgren, Axel Gerdtsson, Dag Halvorsen, Berglind Johannsdottir, Anna K. Jansson, Martin Hellström, Rolf Wahlqvist, Carl W. Langberg, Annika Hedlund, Olof Akre, Ingrid Glimelius, Olof Ståhl, Hege Sagstuen Haugnes, Gabriella Cohn-Cedermark, Anders Kjellman, Torgrim Tandstad

**Affiliations:** a Department of Clinical Science, Intervention and Technology, Division of Urology, Karolinska Institute, Stockholm, Sweden; b Department of Urology, Pelvic Cancer, Karolinska University Hospital, Stockholm, Sweden; c Department of Oncology, Oslo University Hospital, Oslo, Norway; d Department of Urology, Institute of Clinical Science, Sahlgrenska Academy, University of Gothenburg, Gothenburg, Sweden; e Department of Urology, Sahlgrenska University Hospital, Region Västra Götaland, Gothenburg, Sweden; f Department of Urology, Haukeland University Hospital, Bergen, Norway; g Department of Urology, Oslo University Hospital, Oslo, Norway; h Department of Urology, Skåne University Hospital, Malmö, Sweden; i Institution of Translational Medicine, Lund University, Malmö, Sweden; j Department of Urology, St. Olavs University Hospital, Trondheim, Norway; k Department of Pelvic Cancer, Genitourinary Oncology Unit, Karolinska University Hospital, Stockholm, Sweden; l Department of Immunology, Genetics & Pathology, Cancer Precision Medicine, Uppsala University, Uppsala, Sweden; m Department of Radiation Sciences, Oncology, Umeå University, Umeå, Sweden; n The Cancer Centre, Oslo University Hospital, Oslo, Norway; o Department of Oncology, Sahlgrenska University Hospital, Region Västra Götaland, Gothenburg, Sweden; p Department of Molecular Medicine and Surgery, Karolinska Institute, Stockholm, Sweden; q Department of Oncology, Skåne University Hospital, Lund, Sweden; r Department of Clinical Sciences, Lund University, Lund, Sweden; s Department of Oncology, University Hospital of North Norway, Tromsø, Norway; t Department of Clinical Medicine, UIT – The Arctic University of Norway, Tromsø, Norway; u Department of Oncology - Pathology, Karolinska Institute, Stockholm, Sweden; v The Cancer Clinic, St. Olavs University Hospital, Trondheim, Norway; w Department of Clinical and Molecular Medicine, Norwegian University of Science and Technology, Trondheim, Norway

**Keywords:** Germ cell cancer, Metastatic, Retroperitoneal, Robotic surgery, Retroperitoneal lymph node dissection, Seminoma, Testicular

## Abstract

**Background and objective:**

There is an unmet need to avoid long-term morbidity associated with standard cytotoxic treatment for low-volume metastatic seminoma. Our aim was to assess the oncological efficacy and surgical safety of retroperitoneal lymph node dissection (RPLND) as treatment in a population-based cohort of metastatic seminoma patients with limited retroperitoneal lymphadenopathy.

**Methods:**

Sixty-two seminoma patients in Norway and Sweden were included in the cohort from 2019 to 2022. Patients with lymphadenopathy ≤3 cm, having primary clinical stage (CS) IIA/B or CS I with a relapse, were operated with uni- or bilateral template RPLND, open or robot assisted. The outcome measures included surgical complications as per Clavien-Dindo, and Kaplan-Meier survival estimates for 24-mo progression-free survival (PFS) and overall survival (OS).

**Key findings and limitations:**

In the cohort, 33 (53%) had CS I with a relapse during surveillance, six (10%) CS I with a relapse following adjuvant chemotherapy, and 23 (37%) initial CS IIA/B. Metastatic seminoma was verified in 58 patients (94%) with a median largest diameter of 18 mm (interquartile range [IQR] 13–24). Robot-assisted RPLND was performed in 40 patients (65%). Clavien-Dindo III complications were observed in three patients (5%); no grade ≥IV complications occurred. Eighteen patients (29%) received adjuvant chemotherapy after surgery. The median follow-up was 23 mo (IQR 16–30), and recurrence occurred in six patients (10%) after a median of 8 mo (IQR 4–14). PFS was 90% (95% confidence interval: 0.86–1) and OS was 100% at 24 mo.

**Conclusions and clinical implications:**

RPLND as primary treatment is an option for selected low-stage seminomas with a limited burden of disease, showing low complications and low relapse rates, with the potential to reduce long-term morbidity.

**Patient summary:**

In seminoma patients with limited metastatic spread, surgery is a treatment option offering an alternative to chemotherapy or radiation. This paper covers the first 62 patients operated in Norway and Sweden.

## Take Home Message

Retroperitoneal lymph node dissection is a primary treatment option for selected low-stage seminomas with limited retroperitoneal lymphadenopathy, showing low complications and low relapse rates, suggesting the potential to reduce long-term morbidity associated with conventional treatment methods.

## 1 Introduction

Seminomatous germ cell tumors (GCTs) are highly responsive to cisplatin-based chemotherapy as well as to radiotherapy. However, these treatments are linked to several adverse long-term side effects such as cardiovascular, metabolic, renal, pulmonary, neurological, and infertility complications, as well as an increased risk of inducing secondary malignancies [1], [2], [3]. As a result, individuals who have survived metastatic GCTs experience long-term excess mortality [3], [4]. Young patients, expected to have a long lifespan, are predominantly affected.

As metastases of seminoma are often limited to retroperitoneal lymph nodes [5], retroperitoneal lymph node dissection (RPLND) is a compelling treatment modality with a reduced risk of late toxicity-related sequelae. Surgical treatment in metastatic seminoma patients has been evaluated in the prospective trials SEMS, PRIMETEST, and CO-TRIMS [6], [7], [8]. Additionally, several retrospective series have been published from high-volume centers [9], [10]. Performed in specialized surgical units, RPLND is a treatment option associated with relatively low rates of complications and few enduring effects, with the loss of antegrade ejaculation as the most prominent adverse long-term outcome [11], [12], [13]. In theory, RPLND emerges as a favorable treatment option for seminoma patients with low-volume metastatic spread to reduce the extended morbidity and excess mortality associated with chemotherapy or radiotherapy.

**Fig. 1:**
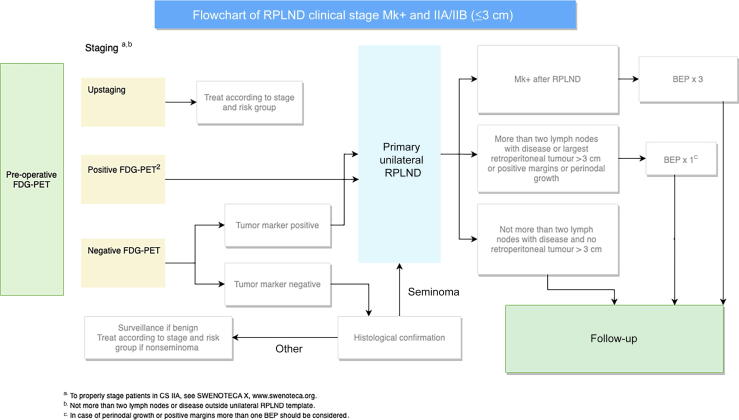
Flowchart from management program SWENOTECA X, 2020, www.swenoteca.org. BEP = bleomycin, etoposide, and cisplatin; CS = clinical stage; FDG-PET = fluorodeoxyglucose positron emission tomography; RPLND = retroperitoneal lymph node dissection; SWENOTECA = Swedish Norwegian Testicular Cancer Group.

The Swedish Norwegian Testicular Cancer Group (SWENOTECA) of oncologists and urologists has, since 1981, provided population-based guidelines and studies for patients with testicular GCTs in Sweden and Norway. The current guidelines, SWENOTECA X, implemented in 2020 recommends primary unilateral nerve-sparing RPLND in modified Royal Marsden clinical stage (CS) Mk+ and IIA-B (≤3 cm in any dimension and no more than two enlarged lymph nodes) [14]. For staging and treatment decisions, fluorodeoxyglucose (FDG) positron emission tomography (PET) is recommended and combined with conventional computerized tomography (CT; see Fig. 1). After RPLND, in case of tumor extension beyond 3 cm and/or two lymph nodes, positive margins, or perinodal growth in the pathology report, adjuvant therapy with one cycle of bleomycin, etoposide, and cisplatin (BEP) is recommended.

In this report, we present the results from the initial cohort of 62 patients who underwent RPLND as primary treatment according to the SWENOTECA X protocol for low-volume metastatic seminoma. The objective was to assess the surgical and oncological outcomes of RPLND as a treatment option for this patient group.

## 2 Patients and methods

### 2.1 Patients and interventions

This is a retrospective analysis of a prospectively enrolled cohort. Adult patients with pure seminoma tumor in the orchiectomy specimen, with CS IIA or IIB by Royal Marsden classification [15], with one to two lymph nodes of maximum 3 cm size (in any dimension) in the retroperitoneum, either at relapse after CS I or at primary metastatic disease, were included from November 2019 to December 2022. Elevated beta human chorionic gonadotropin prior to RPLND was allowed. A preoperative FDG-PET/CT scan was recommended and considered positive if a metastasis exhibited significant hyperbolic metabolism. All patients were discussed at a multidisciplinary tumor board regarding treatment decision. RPLND was performed using either laparoscopic robot-assisted or open surgical technique. The decision regarding the surgical approach was made by the urologist considering the patient’s clinical factors. Surgical templates were used according to the SWENOTECA X guidelines [14]. The right-side template covers the areas below the renal vessels, to the right of the cava, the retrocaval and interaortocaval spaces, and medial to the right ureter. It extends caudally to the aortic bifurcation and to the point where the right ureter crosses the iliac vessels. The left-side template covers the areas below the renal vessels, the retroaortic space, anterior to and to the left of the aorta, and medial to the left ureter. It extends caudally to the aortic bifurcation and to the point where the left ureter crosses the iliac vessels. In addition, both the right and the left template include the ipsilateral testicular vein, from its origin to the profound inguinal orifice. Nerve sparing was at least carried out through a strict template dissection, preserving nerves outside template borders. If deemed possible, a nerve-sparing technique was also employed within templates for all patients. All surgeons had substantial experience in both open and robot-assisted RPLND. Surgery was performed in five tertiary hospitals in Norway and Sweden. The exclusion criteria were elevated alpha-fetoprotein levels, retroperitoneal mass exceeding 3 cm or more than two enlarged lymph nodes on FDG-PET/CT, previous RPLND, former radiotherapy to the abdomen or former chemotherapy other than one adjuvant course of BEP (administered within the SWENOTECA ABC-study [16]), or one course of carboplatin.

Data collection was approved by regional ethics committees.

### 2.2 Protocol for follow-up

The postoperative follow-up included CT of the thorax and abdomen and serum tumor markers at 3 mo, magnetic resonance imaging (MRI) of the retroperitoneum and serum tumor markers at 6 mo, and repeated MRI and tumor markers every 6 mo for the first 2 yr. Thereafter, markers were controlled every 6 mo and MRI of the abdomen was performed annually for 5 yr. X-rays of the thorax were taken at 12, 36, and 60 mo.

### 2.3 Statistical analysis

Clinical parameters collected were age, CS, serum tumor marker levels, radiological examination results, histopathological reports, and adjuvant chemotherapies. Time of follow-up was described using median and interquartile range (IQR). Surgical details included template, side, approach, blood loss, operating time, duration of hospital stay, and postoperative complications. Post-RPLND complications were defined using the Clavien-Dindo classification [17]. The statistical analysis and graphical representations were conducted using STATA v16.1 (StataCorp LCC, College Station, TX, USA). Follow-up started at the time of surgery and ended at the time of relapse or at the last follow-up, whichever came first. A survival analysis was conducted using the Kaplan-Meier method to estimate and compare survival curves between two groups. Subsequently, the log-rank test was applied to assess the statistical significance of observed differences in survival times. The groups were CS, receivers of postoperative adjuvant chemotherapy, and post-RPLND relapse. Kaplan-Meier probability estimates were employed to assess progression-free survival (PFS) and overall survival (OS). At cohort definition, future postoperative adjuvant chemotherapy status was unknown, resulting in Kaplan-Meier curve stratification based on post-time-zero variables, a potential limitation. To assess proportional differences within the individual group of relapses, a one-sided binominal test was utilized for CS, side of testicular tumor, surgical approach, and perinodal tumor growth. Statistical significance was reached at a level of *p* < 0.05 for one- and two-tailed analyses.

## 3 Results

Overall, 62 patients were included in the cohort. Upon enrollment, 48 (77%) patients were classified as having CS IIA and 14 (23%) as having CS IIB. Of these patients, 33 (53%) had a relapse in CS I allocated to surveillance, six (10%) experienced a relapse in CS I following adjuvant chemotherapy, and 23 (37%) had primary metastatic disease CS IIA or B (see Table 1). In 68% of the cohort, one single retroperitoneal lymph node was enlarged. The median time from orchiectomy to RPLND for patients initially classified as having CS I was 17 mo (IQR 14–50). Two patients received bilateral RPLND. One of these patients underwent bilateral approach due to a right-sided orchiectomy tumor and radiographic findings indicating involvement on the left side of the aorta. The other patient had unexpected intraoperative tumor findings in the abdominal region. The median blood loss was 100 ml (IQR 50–195), the median operating time was 139 min (IQR 110–195), and the median duration of hospitalization was 2 nights (IQR 2–6). Complications according to Clavien-Dindo III were observed in three patients (5%) within 30 d, but no grade IV or V complications occurred. Robot-assisted RPLND was performed in 65% of the cohort.

**Table 1:** Patient clinical characteristics and surgical details

Number of patients	62
Age at RPLND (yr), median (IQR)	42 (34–49)
Clinical stage at diagnosis (Royal Marsden), *n* (%)	
CS I	39 (63)
CS IIA	21 (34)
CS IIB	2 (3)
Serum β-hCG at RPLND, *n* (%)	
Normal	57 (92)
Elevated	5 (8)
Value of serum β-hCG (µkat/l), median (range)	0.0 (0.0–316)
Serum LDH at RPLND, *n* (%)	
Normal	38 (61)
Elevated	23 (37)
Not measured	1 (1.6)
Value of serum LDH (IU/l), Norway, median (range)	179 (138–235)
Value of serum LDH (µkat/l), Sweden, median (range)	2.9 (2.0–4.0)
Relapse in clinical stage I, *n* (%)	
CS I in surveillance	33 (53)
CS I following adjuvant treatment	6 (10)
Carboplatin × 1	5
BEP × 1	1
Clinical stage at RPLND (Royal Marsden), *n* (%)	
CS IIA	48 (77)
CS IIB	14 (23)
Positron emission tomography performed (FDG), *n* (%)	61 (98)
Positron emission tomography result, *n* (%)	
Positive	54 (89)
Negative	7 (12)
RPLND template, *n* (%)	
Right side unilateral	23 (37)
Left side unilateral	37 (60)
Bilateral	2 (3.2)
Surgical approach, *n* (%)	
Robot-assisted laparoscopic	40 (65)
Open	22 (36)
Operating time (min), median (IQR)	139 (110–195)
Blood loss (ml), median (IQR)	100 (50–195)
Postoperative complication Clavien-Dindo III, *n* (%)	3 (4.8)
Postoperative hospital duration, nights (IQR)	2 (2–6)

BEP = bleomycin, etoposide, and cisplatin; β-hCG = beta human chorionic gonadotropin; CS = clinical stage; FDG = fluorodeoxyglucose; IQR = interquartile range; LDH = lactate dehydrogenase; RPLND = retroperitoneal lymph node dissection.

Metastatic seminoma was confirmed histologically in 58 patients (94%), two patients (3%) were pN0, one patient (2%) presented with a teratoma, and one patient presented with a nonseminoma other than teratoma (see Table 2). Microscopic perinodal extension of tumor cells was observed in 18 patients (29%). Adjuvant chemotherapy was administered to 18 (29%) of the patients after RPLND, 17 of them receiving one course of BEP. One patient underwent adjuvant chemotherapy on two occasions: a single course of carboplatin following orchiectomy in CS I and 5 yr later one course of BEP after RPLND.

**Table 2:** Histological and clinical outcomes after RPLND

No. of patients	62
Lymph node yield (*n*), median (IQR)	11 (8–16)
Malignant lymph node yield (*n*), median (IQR)	1 (1–2)
Diameter of largest malignant lymph node (mm), median (IQR)	18 (12.5–24)
Histopathological finding, *n* (%)	
Seminoma, pN1	58 (94)
Teratoma, pN1	1 (1.6)
Nonseminoma other than teratoma, pN1	1 (1.6)
Necrosis/fibrosis, pN0	1 (1.6)
Benign, pN0	1 (1.6)
Perinodal growth, *n* (%)	
Positive	18 (29)
Negative	42 (68)
pN0	2 (3.2)
Relapse after RPLND, *n* (%)	6 (9.7)
Inside template	4
Outside template	2
Adjuvant treatment after surgery, *n* (%)	18 (29)
BEP × 1	17 (27)
BEP × 2	1 (1.6)
Indication for postoperative adjuvant treatment, *n*	
Extended no. of malignant lymph nodes	5
Perinodal growth	4
Combination no. of malignant lymph nodes and size >30 mm	4
Other	5
Follow-up (mo), median (IQR)	23 (16–30)

BEP = bleomycin, etoposide, and cisplatin; IQR = interquartile range; RPLND = retroperitoneal lymph node dissection.

In all, six patients have relapsed, and all were treated successfully. One patient initially classified as having CS IIB exhibited two large tumors measuring over 4 cm in the pathology report. The patient was evaluated to still harbor metastatic disease following RPLND and subsequently received three courses of BEP. However, shortly after completing chemotherapy, the patient experienced a new recurrence in the retroperitoneum, and thereafter received paclitaxel, ifosfamide, and cisplatin in two cycles and subsequent high-dose chemotherapy (carboplatin and etoposide). Initially, this patient had a delay in orchiectomy due to personal choice, for 3½ yr from the onset of symptoms. Relapses occurred within the surgical treatment field in four patients (67%). No proportional differences were identified within the relapsing patients when stratifying for CS, surgical approach, or perinodal tumor growth. The only significant proportional difference observed among patients who experienced a relapse after surgery was a left-sided testicular tumor (*p* = 0.03), in addition to the absence of postoperative adjuvant treatment. At the last follow-up of August 2023, all patients with previous recurrence were disease free. Comprehensive outlines of relapses and surgical complications are presented in Supplementary Tables 1 and 2.

**Fig. 2:**
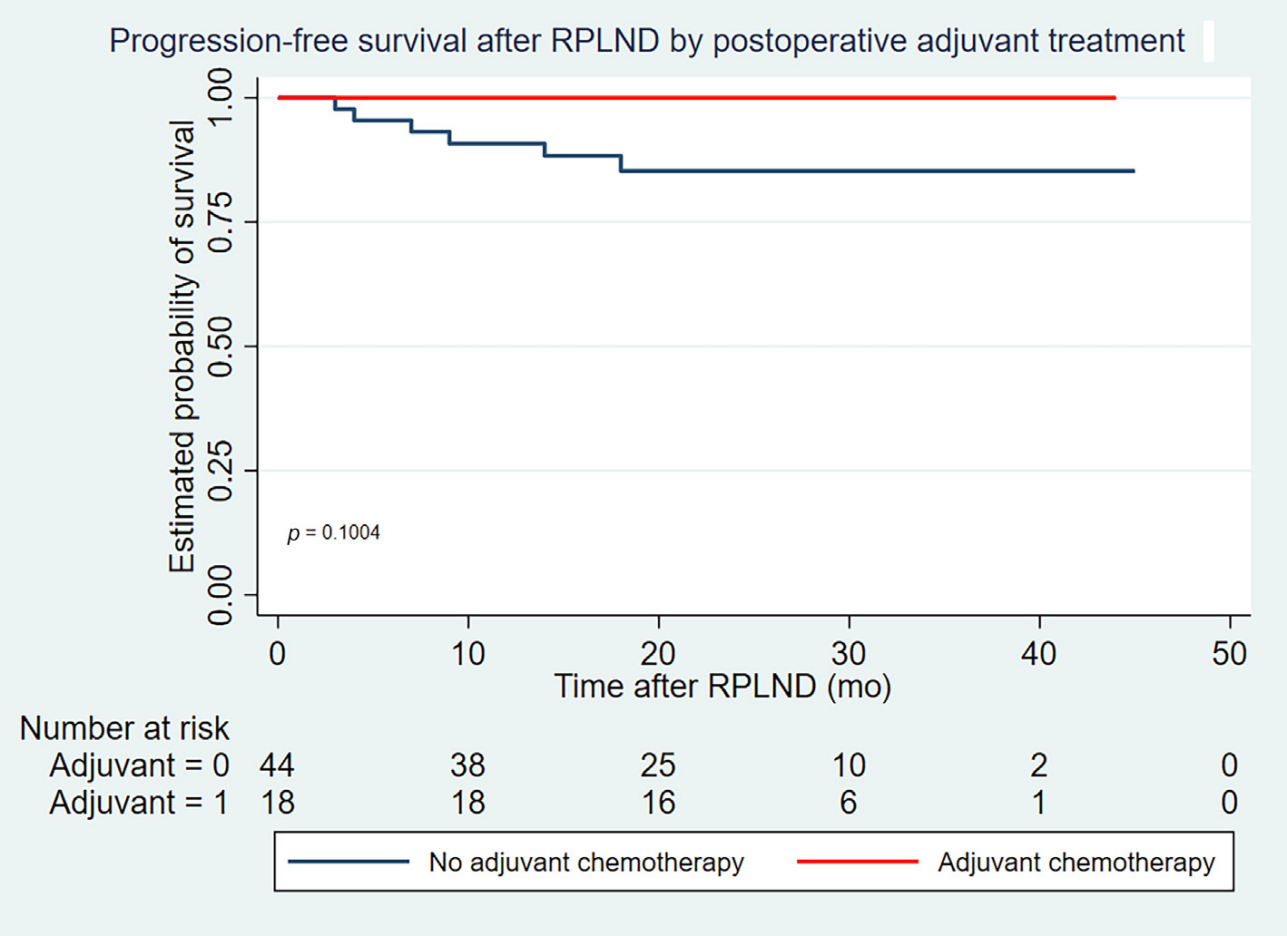
Progression-free survival after RPLND stratified on postoperative adjuvant treatment. RPLND = retroperitoneal lymph node dissection.

PFS at 24 mo was 90% (95% confidence interval [CI]: 0.86–1) and OS was 100%. No significant differences in PFS were discovered when stratifying for CS. A subgroup analysis after surgery demonstrated 100% PFS for the adjuvant-treated group and 86% PFS for patients without adjuvant treatment (95% CI: 0.76–0.96) at 24 mo (*p* = 0.1; see Fig. 2). The median time for follow-up for the 56 patients not experiencing a relapse was 24 mo (IQR 18–30).

## 4 Discussion

In addition to presenting the largest cohort to date, this is also the first report on outcomes for RPLND as the preferred treatment in low-volume seminoma at a population-based level. For CS IIA/B seminomas, radiotherapy has traditionally been the curative treatment option. However, this approach was abandoned in the SWENOTECA guidelines due to a high risk of relapse (10–19%) [5], [18], [19]. For the past decade, the standard treatment for this group has been three courses of BEP or four courses of etoposide and cisplatin. The recurrence rate after first-line chemotherapy in CS IIA/B seminoma has been reported by SWENOTECA to be close to zero [5]. The primary objective of the SWENOTECA X protocol is therefore prioritizing safety and reduction in treatment burden without compromising the survival outcomes. We found the rates of complications and surgical outcomes to be comparable with earlier reported findings in SEMS, PRIMETEST, and CO-TRIMS (see Table 3). However, the recurrence rate was lower: 10% in SWENOTECA, as opposed to 22% in SEMS and 30% in PRIMETEST. When evaluating this finding, one must acknowledge that the time of follow-up is significantly shorter and therefore the rate of recurrence is highly likely to increase to the levels of our colleagues’ reported results. Addressing other variables credible to influence the recurrence rate, the SWENOTECA cohort enforces more restrictive size criteria than PRIMETEST and CO-TRIMS, and one may hypothesize that larger metastases could increase the risk of recurrence. Finally, 29% of the patients treated according to our protocol received postoperative adjuvant chemotherapy (most by one cycle of BEP), which has probably reduced the recurrence rate. The rate of postoperative adjuvant treatment given in SEMS was 2% (carboplatin), and zero in PRIMETEST and CO-TRIMS. None of the six patients with a postoperative relapse in our cohort received adjuvant treatment after RPLND. Other than an overweight for left-sided tumors, we were unable to identify patterns among the limited number of patients who experienced relapse. No associations could be established regarding the initial CS, perinodal extension, or surgical approach. Given that the cohort was established prior to the decision regarding adjuvant treatment, it is important to interpret the Kaplan-Meier analysis within this contextual framework.

**Table 3:** Summary of outcomes in four cohorts of seminoma patients with limited retroperitoneal lymphadenopathy treated with primary RPLND, counts (*n*) and percentage (%)

	PRIMETEST	SEMS	CO-TRIMS	SWENOTECA
Cohort	*n* = 33	*n* = 55	*n* = 30	*n* = 62
Inclusion	Seminoma CS IIA-B Mk- <5 cm	Seminoma CS IIA-B <3 cm	Seminoma CS IIA-B Mk- <5 cm	Seminoma CS IIA-B ≤3 cm, ≤2 nodes
Median follow-up (mo)	32	33	22	23
Robot-assisted laparoscopic RPLND (%)	58	0	10	65
Clavien-Dindo ≥III (%)	12	2	13	5
pN0 (%)	9	16	10	3
Postop adjuvant chemotherapy (%)	0	2	0	29
Recurrence rate (%)	30	22	10	10

CS = clinical stage; RPLND = retroperitoneal lymph node dissection.

Long-term complications, such as the rate of retrograde ejaculation, have not been reported in this article. A more focused study on long-term effects in RPLND patients is currently underway by SWENOTECA.

The preoperative clinical staging of the group was largely coherent with the histopathological findings, with a predominance of corresponding CS IIA and a median tumor size of 18 mm. Eight patients (13%) exhibited postoperative retroperitoneal metastasis of ≥3 cm, but none exceeded 5 cm, which would correspond to CS IIC. The rate of benign lymph nodes (pN0) in this cohort was 3%. One of the two patients displayed necrosis and fibrosis in a retroperitoneal lymph node, suggesting the presence of a prior tumor. The other patient with a benign outcome exhibited an unexpected, undiagnosed primary syphilis infection—a relative rarity in the Scandinavian countries. Radiological findings in this patient revealed a lymph node enlargement mimicking a malignant spread of the previous seminoma in CS I. It is worth noting that all patients in Sweden considered for RPLND undergo evaluation through a national multidisciplinary tumor board attended by urologists and oncologists specialized in germ cell cancer. Similar multidisciplinary conferences are held at a tertiary center level for the Norwegian patients. We believe that this collaborative approach has contributed to the low incidence of benign postoperative outcomes. Moving forward, there are more potential strategies to refine the selection of candidates for surgery. One promising addition is the utilization of the novel biomarker miR-371a-3p, which has demonstrated remarkable performance in detecting germ cell cancer, particularly seminomas [20], [21], [22], [23]. Combining conventional and contemporary biomarkers with PET and expertise consensus can potentially enhance further the precision of selecting seminoma patients for RPLND. Although FDG-PET/CT was a recommended part of the decision procedure, it is still on an experimental level. We intend to provide a detailed report on the PET findings within this patient group at a later date.

A ten-fold increased recurrence rate after surgery compared with conventional systemic therapy, as demonstrated by this report, should be regarded in the context of anticipated considerably fewer long-term sequelae and future risks for these young patients. Prolonged follow-up has not identified any negative consequences resulting from short adjuvant chemotherapy [24]. However, it is important to acknowledge that the cohort’s size is insufficient for drawing any definitive conclusions. Nevertheless, the strengths of this analysis include a homogenous population-based binational cohort, with close coherence to guidelines, treatment centralized to tertiary centers, and no loss to follow-up. Since a metastatic GCT is a rare condition, difficulties collecting enough patients to achieve statistical power or even randomization are evident. The best available strategy seems to be combining findings from prospective trials such as the four discussed here, and continually enrolling patients in prospective cohorts executed by experienced and committed RPLND surgeons as part of a multidisciplinary team. Given these strategies, surgery holds the possibility of evolving into the gold-standard treatment for low-volume metastatic seminomatous GCTs.

## 5 Conclusions

Surgical intervention in the form of RPLND as primary treatment is an option for selected low-stage seminomas with a limited burden of disease, showing fewer complications and low relapse rates. This option carries the potential to reduce long-term morbidity and treatment-related mortality in comparison with radiotherapy and multiple courses of chemotherapy. Our data demonstrated high oncological efficacy and low morbidity. The results are dependent on meticulous staging of patients and the RPLND procedure being performed by an experienced surgeon at a specialized center.

  ***Author contributions*:** Anna Thor had full access to all the data in the study and takes responsibility for the integrity of the data and the accuracy of the data analysis.

  *Study concept and design:* Thor, Cohn-Cedermark, Kjellman, Tandstad.

*Acquisition of data:* Thor, Negaard, Grenabo Bergdahl, Almås, Melsen Larsen, Johannsdottir, Jansson, Hellström, Wahlqvist, Langberg, Hedlund, Glimelius, Ståhl, Haugnes, Cohn-Cedermark, Kjellman, Tandstad.

*Analysis and interpretation of data:* Thor, Cohn-Cedermark, Kjellman, Tandstad.

*Drafting of the manuscript:* Thor.

*Critical revision of the manuscript for important intellectual content:* Thor, Negaard, Grenabo Bergdahl, Almås, Melsen Larsen, Lundgren, Gerdtsson, Halvorsen, Johannsdottir, Jansson, Hellström, Wahlqvist, Langberg, Hedlund, Akre, Glimelius, Ståhl, Haugnes, Cohn-Cedermark, Kjellman, Tandstad.

*Statistical analysis:* Thor, Lundgren, Kjellman, Tandstad.

*Obtaining funding:* Thor, Cohn-Cedermark, Kjellman.

*Administrative, technical, or material support:* Thor, Cohn-Cedermark, Kjellman, Tandstad.

*Supervision:* Cohn-Cedermark, Kjellman, Tandstad.

*Other:* None.

  ***Financial disclosures*:** Anna Thor certifies that all conflicts of interest, including specific financial interests and relationships and affiliations relevant to the subject matter or materials discussed in the manuscript (eg, employment/affiliation, grants or funding, consultancies, honoraria, stock ownership or options, expert testimony, royalties, or patents filed, received, or pending), are the following: Anders Kjellman has received speaker honoraria from Janssen R&D and Astellas; has participated in trials for Astellas, Janssen R&D, Bayer, and Immunicum; and has been on advisory board for Astellas. Ingrid Glimelius has participated in educational events for and has received nonrelated research funding to the department from Jansen Cilag, Takeda, and Lokon Pharma. The remaining authors have no conflicts of interest.

  ***Funding/Support and role of the sponsor*:** This work was supported by grants from the Swedish state under the agreement between the Swedish government and the county councils, the ALF-agreement; from the Karolinska University Hospital; and from the Swedish Cancer Society.

  ***Data sharing statement*:** The data supporting the findings of this report are available upon reasonable request to the corresponding author.
